# Label-Free Surface Enhanced Raman Spectroscopy for Cancer Detection

**DOI:** 10.3390/cancers14205021

**Published:** 2022-10-14

**Authors:** Ertug Avci, Hulya Yilmaz, Nurettin Sahiner, Bilge Guvenc Tuna, Munevver Burcu Cicekdal, Mehmet Eser, Kayhan Basak, Fatih Altıntoprak, Ismail Zengin, Soner Dogan, Mustafa Çulha

**Affiliations:** 1 Department of Genetics and Bioengineering, Faculty of Engineering, Yeditepe University, Istanbul 34755, Turkey; 2 Sabanci University Nanotechnology Research and Application Center (SUNUM), Istanbul 34956, Turkey; 3 Department of Ophthalmology, Morsani College of Medicine, University of South Florida, Tampa, FL 33612, USA; 4 Department of Chemistry, Canakkale Onsekiz Mart University, Canakkale 17020, Turkey; 5 Department of Biophysics, School of Medicine, Yeditepe University, Istanbul 34755, Turkey; 6 Department of Medical Biology, School of Medicine, Yeditepe University, Istanbul 34755, Turkey; 7 Department of General Surgery, School of Medicine, Istinye University, Istanbul 34010, Turkey; 8 Department of Pathology, Kartal Dr. Lütfi Kırdar City Hospital, University of Health Sciences, Istanbul 34865, Turkey; 9 Department of General Surgery, Research and Educational Hospital, Sakarya University, Serdivan 54100, Turkey; 10 The Knight Cancer Institute, Cancer Early Detection Advanced Research Center (CEDAR), Oregon Health and Science University, Portland, OR 97239, USA; 11 Department of Chemistry and Physics, College of Science and Mathematics, Augusta University, Augusta, GA 30912, USA

**Keywords:** SERS, silver nanoparticles, cancer detection, blood, human serum, plasma

## Abstract

**Simple Summary:**

Blood is considered a rich reservoir of biomarkers for disease diagnosis. Surface-enhanced Raman scattering (SERS) is known for its high sensitivity and has been successfully employed to differentiate blood samples from cancer patients versus healthy individuals. Different from previous reports, this study aims at investigating the reliability of the observed results by varying several parameters influencing the observed spectra. Thus, blood taken from 30 healthy individuals as the control group, 30 patients with different types of cancers, and 15 patients with various types of chronic diseases were used in the study. The results revealed that spectral differences in the cancer group was directly related to the presence of cancer-related biomarkers. Although data were obtained from only small group of patients, the recorded sensitivity and specificity values clearly show the power of the technique to detect cancer.

**Abstract:**

Blood is a vital reservoir housing numerous disease-related metabolites and cellular components. Thus, it is also of interest for cancer diagnosis. Surface-enhanced Raman spectroscopy (SERS) is widely used for molecular detection due to its very high sensitivity and multiplexing properties. Its real potential for cancer diagnosis is not yet clear. In this study, using silver nanoparticles (AgNPs) as substrates, a number of experimental parameters and scenarios were tested to disclose the potential for this technique for cancer diagnosis. The discrimination of serum samples from cancer patients, healthy individuals and patients with chronic diseases was successfully demonstrated with over 90% diagnostic accuracies. Moreover, the SERS spectra of the blood serum samples obtained from cancer patients before and after tumor removal were compared. It was found that the spectral pattern for serum from cancer patients evolved into the spectral pattern observed with serum from healthy individuals after the removal of tumors. The data strongly suggests that the technique has a tremendous potential for cancer detection and screening bringing the possibility of early detection onto the table.

## 1. Introduction

Cancer, also called malignancy, is among the most common and deadly disease in the world [1,2]. It arises from the malfunction of certain genes as a result of mutations, ultimately leading to uncontrolled cell proliferation [3]. According to the National Cancer Institute there are more than 100 types of cancer, including breast, abdominal, gastric, liver, intestinal, rectal, colon, skin, lung, prostate cancer, and lymphoma [4]. Cancer diagnosis is a complicated process involving several imaging techniques, molecular tests and finally pathological examination. Cancer symptoms vary depending on its type. After an initial evaluation by a clinician, combinations of imaging techniques and molecular tests are used for specific symptoms. Imaging techniques are used to locate tumorous tissue and determine the size if, is any. The tests involve tissue biopsy followed by histopathological examination, and molecular tests involving body fluids such as blood and urine [5,6,7,8]. Recently, there is a remarkable effort to utilize body fluids for cancer diagnosis, called “liquid biopsy” and refers to the analysis of a liquid specimen obtained in a noninvasive or minimally invasive manner from patients. The reason behind this effort is that they are considered a good biomarker reservoir. However, all bodily liquids, including blood, are very complex mixtures carrying diverse molecular structures, including ions, small molecules, metabolites, proteins, DNA fragments, extracellular vesicles (EVs), and cells and it is quite tedious work to detect a target biomarker in such a complex mixture. Blood also contains numerous cancer-related molecular species including circulating tumor cells (CTCs), circulating tumor DNA (ctDNA), tumor-derived EVs, circulating non-coding RNAs (ncRNA) shed from tumors and their metastatic sites into the blood stream. The assessment of these biomarkers is considered as an effective strategy for cancer screening and early diagnosis [9]. Cancer detection by a minimally invasive way is not only important for diagnosis but also critical to improve prognosis and increase the survival. Although there are screening methods involving imaging techniques and/or tumor marker detection in body fluids, the need for more sensitive, faster and cost-effective alternative approaches is clear [10]. 

Raman spectroscopy (RS) has been investigated for its potential as an alternative technique for cancer diagnosis from cells, tissues or body fluids, such as saliva, urine and blood in the past two decades [11,12,13,14,15,16,17,18,19,20,21]. Due to the fact that a Raman spectrum from a complex mixture can provide critical molecular information about the relative abundance of components in that mixture, the relative compositional changes in the mixture can be monitored. Since all biomacromolecules and molecular structures, such as nucleic acids, proteins, lipids and carbohydrates have their own characteristic Raman spectral fingerprint, in theory it is possible utilize RS to monitor their changes in cells, tissues and body fluids as a result of a disease. However, the use of RS is often limited by its low sensitivity. For instance, the concentration of most metabolites in biofluids; serum, urine or tear is below the detection limit of the technique. However, a phenomenon called Surface-enhanced Raman scattering (SERS), discovered in early 1970s, enhances RS up to 10^14^ times and can be a remedy for this deficiency of the technique by bringing the molecule of interest into contact or very close vicinity of a nanostructured noble metal surface, such as gold or silver [22,23,24,25]. With its extremely high sensitivity, SERS has been employed to solve numerous challenging detection problems in a variety of fields, including biotechnology and medicine [26,27,28,29]. The first SERS report regarding the detection of cancer from blood plasma, investigated nasopharyngeal cancer [30]. Later, the detection of different cancer types with the technique was reported [31,32,33,34,35,36,37,38,39,40,41,42,43,44,45,46,47,48,49]. We also demonstrated that SERS could successfully differentiate cancerous tissues and cells from controls in our previous studies [50,51,52]. Even in a comparison of SERS and Raman spectroscopy for the detection of breast cancer from serum samples found that SERS was more successful [53]. The potential of the technique for cancer and disease detection from liquid biopsies has recently been reviewed [54,55]. Varying SERS substrates and sample preparation strategies were also reported for the screening of several diseases, including cancer [56,57,58]. 

In this study, we investigated the potential of SERS to detect cancer in blood samples by studying a number of parameters, including blood samples from cancer patients with a chronic disease diagnosis. We used a simple sample preparation approach which involved mixing colloidal silver nanoparticles (AgNPs) with blood plasma. As the AgNP colloidal suspension was mixed with the plasma sample, the SERS spectra were influenced through both aggregation status and total available surface area to interact with biological molecular species in plasma. Since blood composition varies throughout the day with diet and disease conditions, the concentration of a biomolecule in plasma may also vary. In order to test such scenarios, we first investigated the effect of AgNP concentration mixed with plasma sample on SERS spectra, then, the concentration effect of small molecules, including arginine, proline, serine, valine, histidine, tryptophan, tyrosine, phenylalanine, urea, mannose, thymine, cytosine, guanine, xanthine, hypoxanthine (Hx), and adenine, and finally, proteins, including human serum albumin (HSA), transferrin (Trf), lysozyme (Lys). Evaluation of the SERS data from these experiments provided extremely useful information about the possible origins of the spectral bands observed on a blood serum spectrum. Then, we showed the discrimination of SERS spectra for serum samples obtained from cancer patients and healthy individuals using principal component analysis and discriminant analysis (PCA-DA), and partial least square discriminant analysis (PLS-DA). In addition, we compared the SERS spectra of serum samples obtained from individuals with chronic diseases and cancer patients. Lastly, we showed that spectral shape of the serum samples collected after tumor removal was somewhere between that of patients before the tumor removal and of the samples from the healthy individuals.

## 2. Materials and Methods

All chemicals were purchased from Sigma-Aldrich (St. Louis, MI, USA). Double-distilled, deionized water was obtained using a Millipore DIRECTQ-UV system. Amicon Ultra-15 centrifugal filter units (3 kDa) were purchased from Merck Millipore (Burlington, MA, USA). Human serum (catalog no H6914) was purchased from Sigma-Aldrich (St. Louis, MI, USA).

### 2.1. Synthesis and Characterization of AgNPs

AgNPs were synthesized using the Lee-Meisel citrate reduction method [59]. Briefly, 180 mg of silver nitrate (AgNO_3_) was dissolved in deionized water and the solution was heated until boiling. Then, 20 mL of a 1% trisodium citrate solution was added dropwise. After 90 min of boiling, suspension was cooled to room temperature. Loss of water due to evaporation was compensated for by adding water to make a 1 L of colloidal AgNP suspension. The concentration of the AgNPs suspension was 1.28 nM and denoted as 1×. AgNPs at concentrations of 64, 32, 16, 8, 4, and 2 times were prepared by centrifugation and removal of a portion of the supernatant. These concentrates are denoted as 64×, 32×, 16×, 8×, 4×, 2×. pH values for the prepared colloid samples were between 7 and 8. The synthesized AgNPs were characterized and monitored for their stability using UV-visible (UV/Vis) spectroscopy and dynamic light scattering (DLS) once a week over seven months. The synthesized colloidal suspension was stored in a glass bottle at 4 °C and manually shaken twice a week to prevent AgNPs sedimentation since changes in particle size and charge may result in different SERS spectral patterns. Figure S1A shows the UV/Vis spectrum of the citrate-reduced AgNPs over the seven months. Maximum absorption of the spectrum was 405 nm, corresponding to an average size of 30 nm. The zeta potential is a key indicator of the stability of the AgNP colloidal suspension. Figure S1B shows that the zeta potential of the AgNPs was around −40 mV for this time period. The stability behavior of the AgNP colloids depending on the zeta potential was shown to have a good stability.

### 2.2. Collection and Preparation of Human Blood Plasma Samples 

The human blood experiment study complied with the Declaration of Helsinki, and the study protocol was approved by the Yeditepe University Ethical Committee (KAEK No. 1523). All donors signed an informed consent form before enrolling in the study and participants were all older than 18 years old. Demographic, clinical and laboratory data for each patient were recorded from the participants’ files with the permission of participants. After 12 h of overnight fasting, blood samples were collected from 30 healthy individuals (non-smokers, 18 male and 12 female donors, mean age: 54.9, ±SD: 11.2, median age: 56) as the control group, 30 patients with different types of cancer, and 15 patients with various types of chronic diseases. The detailed information of study population (e.g., age, gender, disease and histopathological stage) is summarized in Tables S1 and S2. All samples were collected at either Kartal Dr. Lütfi Kırdar Hospital, Istanbul, or Sakarya University Hospital, Sakarya, Turkey, and then processed at Yeditepe University. To detect whether the patient had a tumor, the tissue specimens removed during surgery were sent to pathology for histopathological analysis. Then, the samples were examined macroscopically by light microscopy after staining. A portion of tumor tissue samples were stored at −80 °C until further used. The rest of the tumor tissues were fixed with 10% phosphate-buffered formalin solution for 6–48 h. The blood samples were drawn and kept at room temperature for about 60 min, and then centrifuged at 4500 rpm for 10 min. After centrifugation, serum was obtained and was then transferred to clean DNAse, RNAse-free tubes. The samples were stored at −80 °C prior to SERS measurements. The filtered human serum was obtained using a filtration unit with a 3 kDa cut-off point. Since commercial filters may contain glycerol, filters were cleaned just before serum filtration following the protocol suggested by Bonnier et al. [60]. Then, 5 mL of commercial human serum placed into the filter unit was centrifuged at 4000 *g* for 1 h.

### 2.3. SERS Measurements and Data Analysis Methods

SERS spectra were collected using a Raman microscopy system (InVia Reflex, Renishaw, UK) equipped with an 830 nm diode laser and 50× objective. The system was automatically calibrated against a silicon wafer peak at 520 cm^−1^ each time before experiments. Laser power was 1.5 mW. Exposure time was 10 s for all measurements. For the SERS experiments, 5 μL of whole serum (WS) samples from donors, 5 μL of filtered commercial serum samples (FS), and 5 μL of several small molecules (2 mM) such as arginine, proline, serine, valine, histidine, tryptophan, tyrosine, phenylalanine, urea, mannose, thymine, cytosine, guanine, xanthine, hypoxantine (Hx), and adenine were mixed with the same concentration of AgNPs colloidal suspension (64×). Then 2 μL of each mixture was spotted onto a CaF_2_ slide and dried at room temperature for SERS measurements. Please note, the final concentration of AgNPs and molecules decreased by a factor of two due to the mixing (i.e., AgNPs concentration became 32× and molecule concentrations became 1 mM after mixing). In the rest of the manuscript, final concentrations after mixing are used. Ten spectra were acquired from the middle region of three different droplets for each sample. The SERS spectra were smoothed, baseline corrected, and normalized using instrument’s Wire 4.1 software (Renishaw plc, UK). Each acquired SERS spectrum had 1715 data points (dimensions) between 400 and 1800 cm^−1^ (spectral resolution was 0.816 cm^−1^). Therefore, dimension reduction was necessary to analyze and differentiate the acquired spectra. Multivariate analysis methods, PCA-DA and PLS-DA, were used for these purposes. Both techniques combine dimension reduction with discriminant analysis into one algorithm. While the PCA-DA technique uses principal components, PLS-DA uses latent variables for discrimination and classification. Both techniques have been widely used for SERS studies [61,62,63,64]. Multivariate data analyses (PCA-DA and PLS-DA) were performed using Classification Toolbox version 4.2 [65] in the MATLAB environment

## 3. Results 

### 3.1. Evaluation of the Influence of Silver Colloid Concentration on the Enhancement Effect

It is well known that reproducibility is the most fundamental issue in a SERS experiments. Thus, a number of parameters, including AgNP colloidal suspension stability by time and the concentration of AgNPs in the final serum–AgNP mixture, throughout the study was evaluated to assure the reproducibility of the SERS spectra. As mentioned above, the stability of the AgNP suspension was assured during the study. Blood serum contains salt ions which trigger AgNP aggregation regardless of proteins content. For surface-enhanced Raman scattering (SERS), molecules must be within close proximity of the nanoparticles (1–4 nm). When two nanoparticles are close a so called “hot spot” volume occurs during laser illumination, and when molecules are in this hot spot, their SERS enhancement increases. SERS enhancement of proteins are much lower than that of small molecules. When proteins fill the gaps among AgNPs and thus increase the distance between AgNPs, SERS enhancement of serum molecules cannot occur [66]. Although serum protein content can be reduced, we decided to use serum without applying a protein removal procedure to prevent possible loss of biomolecular species that might have a role in the observed SERS spectra. On the other hand, a high protein content of serum prevents AgNP aggregation which results in low intensity signals [66] and causes uneven distribution of AgNP aggregates in the dried droplet area. Therefore, a significant number of AgNPs must be present to obtain a uniform distribution of AgNPs in the spectrum-acquired region of the droplet area to achieve good reproducibility. In order to find an optimum colloidal AgNP concentration, a volume of AgNP colloidal suspension with a concentration of 32×, 16×, 8×, 4×, 2×, 1× and 0.5× (final concentrations after mixing) was mixed with both whole (unfiltered) serum (WS) and filtered serum (FS) for SERS measurements. Figure S2 shows that the use of 32× and 16× AgNP concentrations produced the most reproducible spectra for WS. As seen in Figure S2, as the AgNP concentration in the final mixture decreased, the spectral intensity decreased and the spectral noise increased. To demonstrate the protein effect on the SERS spectra, the same SERS experiments were repeated for FS. As seen in Figure S3, from 32× AgNP to 0.5× AgNP concentrations the intensity of the acquired spectra only decreased by 50% and the spectra did not become noisy. Considering the results discussed so far, the 32× AgNPs concentration was the best choice to use in the experiments. Another important point was homogeneity of the AgNPs in the dried droplet area. To understand this, we mapped the middle of a dried droplet of 32× AgNP colloid and WS mixture as in Figure S4. The deposit had a doughnut shape due to protein content being swept to the peripheries during drying as a result of the “coffee-ring phenomenon”. A 600 μm × 600 μm region in the middle of the droplet was mapped with a 5 μm step size and intensity distribution at 638 cm^−1^ was shown as a color map. As seen in Figure S4, the intensity distribution was nearly uniform. The results show that a 32× AgNPs concentration was the best choice for the SERS experiments. 

### 3.2. SERS Spectra of Small Biomolecules in the Serum

**Figure 1:**
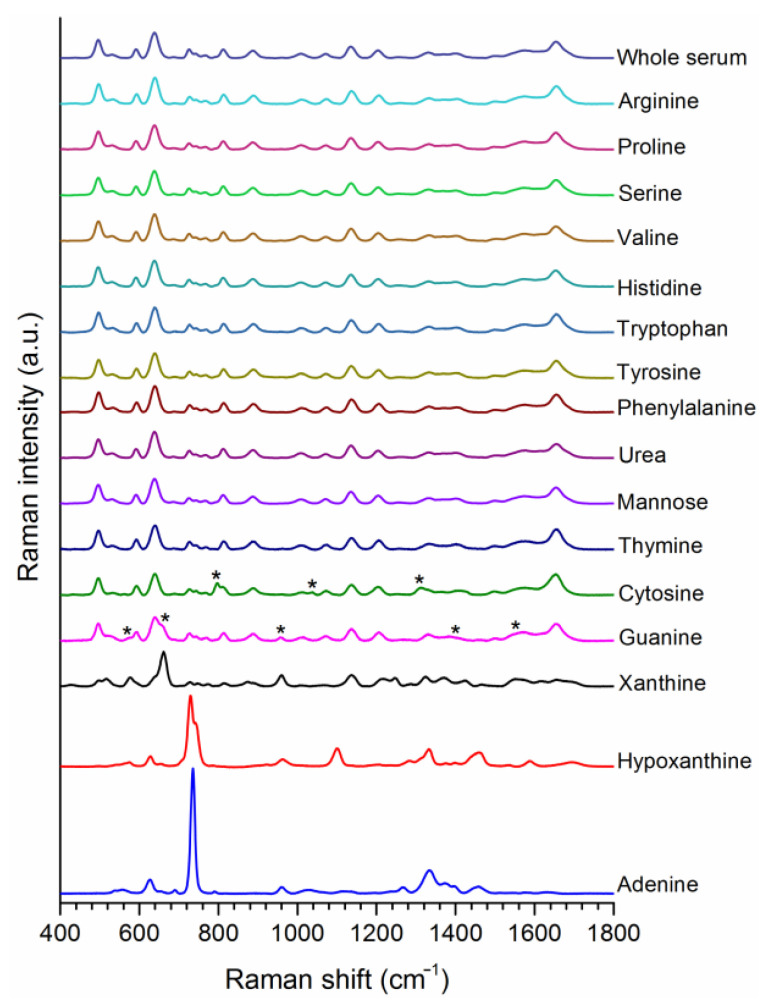
SERS spectra of whole serum (WS) after spiking with the indicated molecules (1 mM) found in blood serum. Star symbols indicate the bands contributed to the serum SERS spectra by the spiked metabolites.

Figure S5 shows SERS spectra of small molecules, whose final concentrations after mixing with AgNPs were 1 mM, which is a much higher value than their physiological serum concentrations in general. It is clearly seen in Figure S5 that only tyrosine, tryptophan, and phenylalanine had the potential to affect the spectral shape among the amino acid samples. Histidine made a partial contribution; however, most of the bands were still of the citrate molecules on the surface of AgNPs. Urea did not make noticeable changes on the spectral shape. For urea, the only difference was the emergence of a band at around 1000 cm^−1^. On the other hand, nucleobases (adenine, thymine, cytosine, and guanine) exhibited completely visible SERS bands, along with purine derivatives xanthine and hypoxanthine. Considering these results, we inferred that if a molecule’s SER bands were not visible at this high concentration when it was mixed with AgNPs, it was very unlikely that it could contribute to the spectral shape of serum. In order to prove this, the same molecules were spiked into commercial human serum (WS) at the same concentration and their spectra were acquired, as shown in Figure 1. This time, none of the amino acids made any change to the spectral shape of WS. Urea and mannose also did not make contributions as expected. In most of the previously published articles, mannose has been reported as the reason for bands at 1095 and 1135 cm^−1^, but we showed here this was not probable. Among nucleobases, cytosine and guanine added some bands to the serum spectra, which means that they could contribute to the serum spectrum if their concentrations in blood changed significantly. The effects of xanthine were more evident than cytosine and guanine. Lastly, we can say that hypoxanthine (Hx) and adenine completely changed the spectral shape of serum.

Other than the studied metabolites, lactic acid is heavily produced by cancer cells, which is known as the Warburg effect in oncology. Lactic acid does not contribute to the SERS spectra of serum in the presence of other molecules that have a higher affinity to the silver surface and higher Raman scattering, such as uric acid, hypoxanthine, and other ring structure possessing amino acids or molecules. Therefore, lactic acid would not be much concern in our experiments and results.

### 3.3. Comparison between Whole Serum (WS) and Filtered Serum (FS)

**Figure 2:**
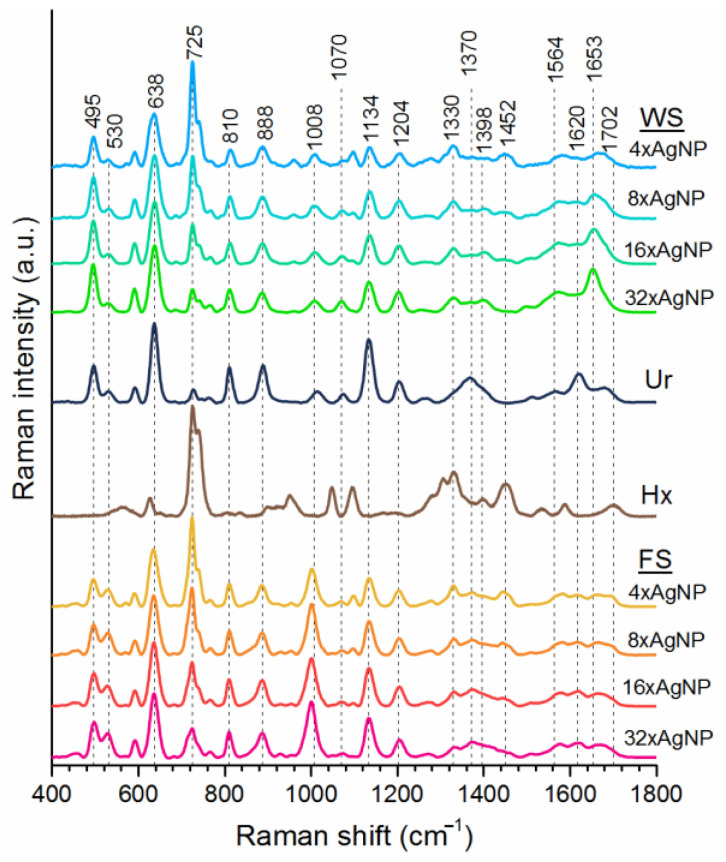
Comparison of SERS spectra for uric acid (Ur), hypoxanthine (Hx), whole serum (WS) and filtered serum (FS) with increasing AgNP concentrations.

According to the Serum Metabolome Database and a related article, there are at least 4651 small molecule metabolites in serum [67]. Regarding proteins, as of 2014, plasma levels of more than one hundred proteins have been estimated [68]. Considering the number, it is obvious that many metabolites and proteins possess an affinity for the silver surface and compete with each other to interact with AgNP surface after serum and AgNP colloid mixing. At low AgNP concentrations, molecules possessing a higher affinity bind to the AgNP surface. Other molecules gain the opportunity to bind as the AgNP concentration is increased. Therefore, at each AgNP concentration, the number and type of molecules under the impinging laser changes, resulting in different spectral outcomes. Monitoring changes in spectral shape can be extremely useful in elucidating the origins of SERS spectra of complex biofluids. By comparing the intensity and patterns of the SERS bands at different AgNP concentrations, better band assignment can be done. To the best of our knowledge, this kind of approach is the first of its kind in the literature. Figure 2 shows SERS spectra of commercial WS and FS at four different AgNP concentrations. Uric acid (Ur) and Hx spectra were also added to the figure because they have some common bands with serum spectra. At a first glance, it is seen that there were dramatic pattern differences among spectra collected using different AgNP concentrations. For example, the intensity of the band at 638 cm^−1^ increased but the intensity of the 725 cm^−1^ band decreased with each two-fold AgNP concentration increment for both WS and FS. Along with the increase in the 638 cm^−1^ band, intensity of 495, 530, 1134 and 1204 cm^−1^ bands increased slightly. On the contrary, intensity of bands at 1330 and 1452 cm^−1^ decreased. Comparing these changes to the spectra of Ur and Hx, it could be said that Ur features replaced Hx features as the AgNP concentration increased. On the other hand, considering the differences in other the bands between serums and these two purine derivatives (1008 cm^−1^, 1370 cm^−1^, 1670 cm^−1^) let us conclude that they are major determinants, but not the only determinants, in the final shape of the serum spectrum. Moreover, the width of the 638 cm^−1^ band is different in the Ur spectrum than the serum spectra, and this reveals the effects of some unknown molecules to the spectral shape. We can attribute the 725 cm^−1^ band in the serum spectrum to Hx because of the presence of a shoulder centered at 744 cm^−1^. Hx has ketonic and enolic forms. At low pH values, it is in ketonic form, and therefore, only the 725 cm^−1^ band can be observed. At high pH values, Hx is in its enolic form and only the 744 cm^−1^ band is observable. At physiological pH values both forms coexist and thus we saw both bands.

Further examination of the spectra in Figure 2 shows us the spectral differences between WS and FS. Differences in the ratio of 495 cm^−1^ and 530 cm^−1^ bands, the intensity of the 1008 cm^−1^ band, and the shape of the 1250–1500 cm^−1^ and 1500–1750 cm^−1^ regions can be seen at first sight. In addition, the 1008 cm^−1^ band is shifted to 1001 cm^−1^. These differences show the importance of proteins on the final shape of WS spectrum. Proteins create molecular crowding on AgNP surfaces and can cause orientation changes of molecules on the AgNP surface. In addition, some proteins bind to the AgNP surface and increase the level of competition for AgNPs. 

### 3.4. Evaluation the Effects of Proteins on Serum SERS Spectra

**Figure 3:**
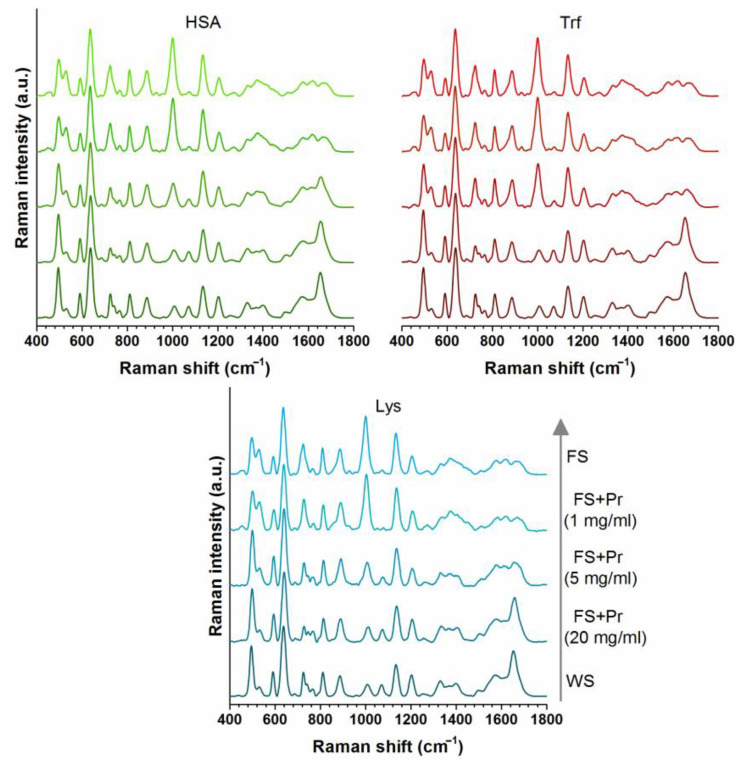
Changes in SERS spectra of free serum (FS) when spiked with different concentrations of HSA, Trf, and Lys. Pr denotes protein. FS + Pr (1 mg/mL) denotes free serum containing protein at 1 mg/mL concentration after mixing with AgNP colloid. Final concentration of AgNP colloid in the mixtures was 32×.

Figure 3 demonstrates that the effect of proteins on serum SERS spectra by using three different model proteins HSA, the most abundant protein, transferrin (Trf), the third most abundant protein in blood serum, and lysozyme. Lysozyme (Lys) has an overall positive charge at physiological pH (pI 11.3), therefore it was chosen to be a model for positively charged proteins in serum. Total protein concentration in serum is in the range of 60–85 mg/mL. In order to mimic this concentration, and to be able to monitor spectral changes due to changes in protein concentration, we spiked FS with proteins at a maximum concentration of 40 mg/mL (please note that this value lowers to 20 mg/mL upon mixing with 64× AgNPs, as shown in Figure 3). When the spiked protein concentration was 1 mg/mL, spectra were similar to FS. A five-fold increase in protein concentrations resulted in significant spectral changes. On the other hand, there were some differences among HSA-, Trf-, and Lys-spiked serum spectra reflecting the effects of each protein type. After a further four-fold increase, the spectra of protein-spiked FS samples became extremely similar to the spectrum of WS acquired using 32× AgNPs (final concentration) regardless of protein type. These effects of high protein concentration on spectral changes need further investigation; however, this is not within the scope of this manuscript. 

### 3.5. SERS Spectra of Healthy vs. Patients

**Figure 4:**
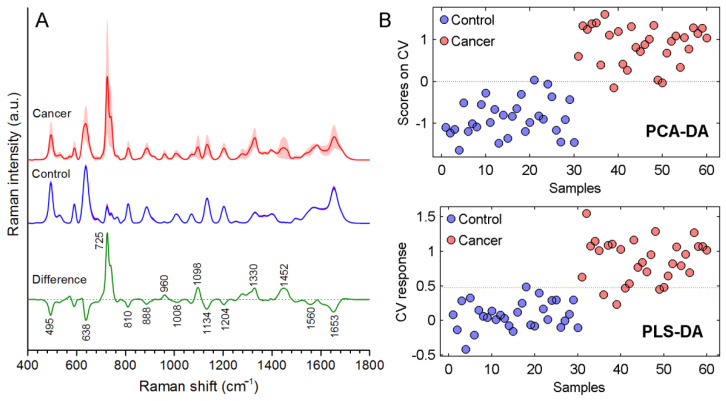
(**A**) SERS spectra of serum samples of cancer patients, of healthy subjects (control) and the difference spectrum of the two groups. (**B**) PCA-DA and PLS-DA scores of each spectrum.

Figure 4 shows the mean SERS spectra of serum samples for 30 cancer patients and 30 healthy subjects (control), along with their differences. Both spectra possessed similar features with commercial WS spectra presented in Figure 2. The spectrum of the control group was similar to the WS spectrum obtained using 32× AgNP, whereas the spectrum of the cancer group was similar to the WS spectrum obtained using 4× AgNPs. It is evident from eight times less AgNP concentration that relative concentrations of the metabolites possessing a higher affinity to the AgNP surface and of the metabolites possessing a larger Raman cross section were higher in the serum of cancer patients. By observing the bands at 725 cm^−1^, 1330 cm^−1^, and 1452 cm^−1^ in the difference spectrum, it can be assumed that one of these metabolites was Hx. On the other hand, the decrease in the intensities of the bands at 495 cm^−1^, 638 cm^−1^, and 830 cm^−1^ shows the decrease in Ur concentration relative to the other metabolites in the cases of cancer disease. What is seen more in Figure 4 is that the standard deviation of the control group spectrum was very low and barely discernible, whereas the standard deviation of the cancer group spectrum was high. For healthy people, the relative concentrations of the metabolites that impact the serum SERS spectrum is about the same; however, for cancer patients, the relative concentrations of the serum constituents are quite variable due to heterogeneity in cancer. A detailed comparison of the cancer and control SERS spectra was performed by statistical analysis on the intensity values of the eight significant SERS bands (Figure S6). During analysis, a Shapiro–Wilk test was applied to check for normality. Since intensity distributions of the eight bands for cancer group were not normally distributed, a Man–Whitney U test was applied to check whether differences in intensity distributions of the two groups were statistically significant. It was found that the *p* value was below 0.05 for each test, showing significant difference between the two groups.

Further data analysis was performed using multivariate statistical analysis tools. Since these types of data analyses can give optimistic results, two different tests (PCA-DA and PLS-DA) were applied to verify the statistical difference between the spectra of cancer group and of control group. Leave-one-out cross validation was done for each test. With PCA-DA, discrimination of the cancer group from the control group was realized with an accuracy of 95%, a sensitivity of 90%, and a specificity of 93%. PCA-DA scores for each spectrum are shown in Figure 4B. The two classes were separated well. With PLS-DA, diagnostic accuracy was 95%, along with 90% sensitivity and 100% specificity. PLS-DA scores for each spectrum can also be seen in Figure 4B. 

Discrimination of SERS spectra of cancer serum samples from control serum samples was not enough to prove that spectral differences were due to cancer. Therefore, we acquired SERS spectra for serum samples of 16 subjects having various types of chronic diseases (Table S2) and compared them to the control group and cancer group (Figures S7 and S8). It was found that the mean spectrum and standard deviation was about the same as that of the control group. Discrimination of the chronic disease group (non-cancer group) from the control group using PCA-DA resulted in 56% sensitivity, 83% specificity, and 74% accuracy. The much higher specificity was due to the higher sample size in the control group. With PLS-DA, this time sensitivity was 67%, specificity was 73%, and accuracy was 71%. Low sensitivity in both tests was due to spectral resemblance between the two groups. On the other hand, the difference spectrum in Figure S7 shows differences between the non-cancer and control groups. The causes of these differences may include inflammation which may accompany chronic diseases and cancer disease, but this requires further investigations and is not within the scope of this manuscript. During discrimination of the cancer group from the chronic disease group, 93% sensitivity, 88% specificity, and 91% accuracy were obtained with PCA-DA. PLS-DA analysis gave similar results: 93% sensitivity, 94% specificity, and 93% accuracy. All multivariate analyses among the three groups showed that spectral differences in the cancer group can be related directly to the presence of cancer disease.

**Figure 5:**
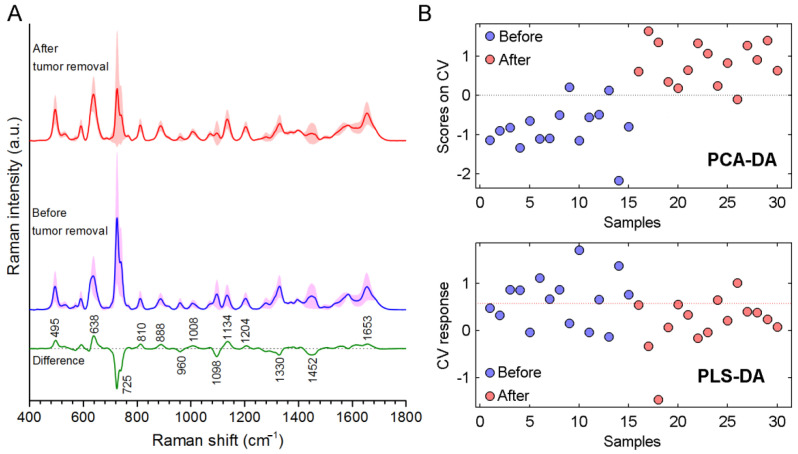
(**A**) SERS spectra for the serum samples of the cancer patients collected before and after tumor removal, and their difference spectrum. (**B**) PCA-DA and PLS-DA scores for each spectrum.

Lastly, we compared SERS spectra of the serum samples obtained from 16 patients before and after tumor removal (Figure 5A). As seen in the figure, mean intensities of 495, 638, 810, 888, 1134 and 1653 cm^−1^ increased after tumor removal. On the other hand, mean intensities of 725, 960, 1098, 1330 and 1452 cm^−1^ decreased. Compared to the difference spectrum in Figure 4, it was seen that changes of the bands were in opposite directions this time. After tumor removal, the concentration of the serum constituents started to return to the normal values, and these changes could be easily seen in the acquired SERS spectra. Figure 5B shows that discrimination data of the serum samples of the cancer patients collected before and after tumor removal. Discrimination after tumor removal samples from before tumor removal samples using PCA-DA was accomplished with 80% sensitivity, 67% specificity, and 71% accuracy. PLS-DA analysis resulted with 87% sensitivity, 60% specificity, and 73% accuracy. Although data were obtained from only 16 patients, the recorded sensitivity and specificity values clearly show the power of the SERS technique.

## 4. Discussion

The spectral reproducibility in a SERS measurement is a key factor and it should be minimized. When the number of molecular species in a complex biological sample is considered, a large SERS substrate surface area is required. Thus, AgNP colloidal suspension concentrations ranging from 32× to 0.5× with both whole (unfiltered) and filtered serum (FS) were evaluated for the best SERS performance and the results revealed that the best concentration was 32× for a reproducible SERS measurement. The other complex and difficult to answer question is the molecular origin of the bands observed on the blood plasma spectrum and the factors its influencing spectral features. Although this is not an easy task, we evaluated a number of possible molecular structures by spiking serum samples to observe their possible contributions. There are at least 4651 small molecule metabolites in serum. In addition, more than one hundred proteins in plasma have been estimated [26,27,67,68,69]. SERS band assignments for both serum and plasma relies mostly on earlier Raman spectra reports of tumor tissues and biological molecules. On the other hand, it is well known that Raman and SERS spectra of biological structures are rather different. Therefore, the band assignment for SERS spectra of any material of interest should be done by SERS acquisition of the molecules in that material. Thus far, only one article has discussed the weight of the bands of two molecules (i.e., uric acid (Ur) and Hx) in serum SERS spectra [40]. In order to fill this gap, we acquired SERS spectra of molecules reported in literature thought to influence the spectral shape of serum using AgNPs. Blood serum contains numerous type of metabolites and proteins, and many of them possess an affinity to the metal silver surface. This creates competitions among them to interact with the AgNPs after mixing. Molecules possessing a higher affinity cover the AgNP surface at low AgNP concentrations. Other molecules can bind to the AgNPs as the AgNP concentration is increased. As a result, the amount and kind of molecules under the laser illumination changes at different AgNP concentrations, which, in turn, results different spectral outcomes. Investigation of these spectral shape changes can be extremely helpful to unravel the origins of SERS spectra of complex biofluids. Better band assignments can be done by comparing the intensities and spectral patterns of the SERS bands at different AgNP concentrations. To the best of our knowledge, this kind of approach is the first of its kind in the literature. A number of small biological molecules at high (1mM) concentrations were tested for their potential contribution to the overall spectrum of a plasma sample. The results showed that only the tested nucleobases and purine derivatives; adenine, thymine, cytosine, guanine, xanthine and Hx resulted with SERS spectra. Thus, these molecules with potential contributions to spectra were spiked into commercial human serum and the resultant SERS spectra recorded. The amino acids, urea and mannose did not make any changes on the spectral pattern of WS. The bands at 1095 and 1135 cm^−1^ are attributed to mannose in the literature but this was not the case in our study. The data suggests that among the nucleobases only cytosine and guanine have the potential to contribute to the overall spectrum depending on their concentration in the blood. The effects of the xanthine were more evident than cytosine and guanine. Lastly, we can say that Hx and adenine completely changed spectral shape of serum.

As soon as the AgNPs were mixed with a serum sample, ionic and molecular species, and proteins adhere to the surface depending on their affinity forming a molecular/ionic layer, similar to a protein corona. The molecules that crowd around the AgNPs can have a strong influence on the overall spectrum. The results clearly indicate that the protein content influences the spectral shape. The protein content is obviously influenced by the molecular crowding on the AgNP surface and can cause orientation changes of molecules on the AgNP surface. 

Finally, SERS spectra of serum samples from 30 cancer patients and 30 healthy subjects were compared. The most staggering observation was the variation difference in the average spectrum of each group. The standard deviation was higher in serum samples from the cancer patients. This suggests that certain molecular species are influencing the overall spectral outcome indicating that the molecular species related to the cancer adhere to the AgNPs or influence the molecular composition of the molecular layer on the surface. Further, comparison of the average spectrum for blood samples from cancer patients to the spectrum obtained for the serum from chronically ill patients revealed the discrimination power of this technique. In the final scenario, the average SERS spectra for serum samples obtained from 16 patients before and after tumor removal was compared and found that removal of the tumor can be detected with very good sensitivity and specificity, clearly demonstrating the power of the SERS technique. Although the sample size was small, results were satisfactory and showed that the SERS technique coupled with multivariate analysis has great potential to be used in clinics in near future.

The study gives an insight into the nature of the observed serum SERS spectra with the aim of cancer detection and screening. The complexity of blood makes it very difficult to discern the contributing molecular species to the overall spectra but as evidenced from this study, there are certain cancer-related metabolites that cause the differences in the observed spectra. The next questions in such cases are what the contributing molecular species are to the spectrum, and which of them have an affinity for AgNP surfaces. 

## 5. Conclusions

Since metabolites in blood serum have different affinities to the AgNP surface and possess different Raman cross sections, the spectral shape of blood serum spectra changes when different AgNP concentrations were used to acquire SERS spectra. Therefore, the use of different AgNP concentrations to investigate the origins of blood serum spectrum provides much more information than using only one AgNP concentration. We think this is also valid for other bodily fluids. Results show that most of the molecules reported in the literature for tentative band assignments for blood serum spectrum actually have no effect on the spectral shape. Furthermore, although Ur and Hx bands are dominant in serum spectra, the final shape of the spectrum is determined by other metabolites and proteins. Future work is necessary to discern other potential contributing molecules. Spectral shape of the serum spectra of healthy subjects and patients with chronic diseases was about the same. On the other hand, spectral patterns changed in the case of cancer. Especially, Hx signals are increased. Moreover, it was observed that the spectral shape for the serum from cancer patients started to resemble that of healthy subjects after tumor removal. This finding shows that SERS can be a useful tool to monitor changes in blood serum composition after tumor removal and the assessment of the success of tumor surgeries.

## Data Availability

The data presented in this study are available in this article (and Supplementary Materials).

## Supplementary Materials

The following supporting information can be downloaded at: https://www.mdpi.com/article/10.3390/cancers14205021/s1, Figure S1: (A) UV/Vis spectra of AgNP colloid over seven months (a total of 28 spectra). (B) Zeta potential of AgNP over seven months. Error bars represent standard deviations of three measurements; Figure S2: SERS spectra of three dried droplets of whole serum (not filtered) samples with increasing AgNP concentrations; Figure S3: SERS spectra of three dried droplets of filtered serum samples with increasing AgNP concentrations; Figure S4: Mapping the middle of a dried droplet. Heat map of the intensity of 638 cm^−1^; Figure S5: SERS spectra of some molecules found in blood serum and SERS spectrum of AgNPs; Figure S6: Box plots of the intensity values of the eight significant SERS bands. The blue line and the green line within each box represent median and mean, respectively. Whiskers represent the 1.5-fold interquartile range; Figure S7: (A) SERS spectra of serum samples of patients with chronic disease (Non-cancer), of healthy subjects (Control) and the difference spectrum of two groups. (B) PCA-DA and PLS-DA scores of each spectrum; Figure S8: (A) SERS spectra of serum samples of patients with chronic disease (Non-cancer), of cancer patients (Cancer) and the difference spectrum of two groups. (B) PCA-DA and PLS-DA scores of each spectrum; Table S1: Demographics of the cancer patients; Table S2: Demographics of patients with chronic diseases.
