# Supplementary material for: Label-Free Surface Enhanced Raman Spectroscopy for Cancer Detection
Source: Cancers (Basel). 2022 Oct 14;14(20):5021. doi: 10.3390/cancers14205021 (PMC9600112; doi:10.3390/cancers14205021)
Supplement: Supplementary file 1 [file cancers-14-05021-s001.zip › cancers-1901927-SI.pdf]

Label-free surface enhanced Raman spectroscopy for cancer detection

Ertug Avci [1], Hulya Yilmaz [2], Nurettin Sahiner [3,4], Bilge Guvenc Tuna [5], Munevver Burcu Cicekdal [6], Mehmet Eser [7], Kayhan Basak [8], Fatih Altıntoprak [9], Ismail Zengin [9], Soner Dogan [6] and Mustafa Çulha [2,10,11,*]

[1] Department of Genetics and Bioengineering, Faculty of Engineering, Yeditepe University, Istanbul 34755, Turkey
[2] Sabanci University Nanotechnology Research and Application Center (SUNUM), Istanbul 34956, Turkey
[3] Department of Ophthalmology, Morsani College of Medicine, University of South Florida, Tampa, FL 33612, USA
[4] Department of Chemistry, Canakkale Onsekiz Mart University, Canakkale 17020, Turkey
[5] Department of Biophysics, School of Medicine, Yeditepe University, Istanbul 34755, Turkey
[6] Department of Medical Biology, School of Medicine, Yeditepe University, Istanbul 34755, Turkey
[7] Department of General Surgery, School of Medicine, Istinye University, Istanbul 34010, Turkey
[8] Department of Pathology, Kartal Dr. Lütfi Kırdar City Hospital, University of Health Sciences, Istanbul34865, Turkey
[9] Department of General Surgery, Research and Educational Hospital, Sakarya University, Sakarya 54100, Turkey
[10] The Knight Cancer Institute, Cancer Early Detection Advanced Research Center (CEDAR), Oregon Health and Science University, Portland, OR 97239, USA
[11] Department of Chemistry and Physics, College of Science and Mathematics, Augusta University, Augusta, GA 30912, USA
* Correspondence: mculha2@gmail.com or mustafa.culha@sabanciuniv.edu or mculha@augusta.edu

Supplementary Materials

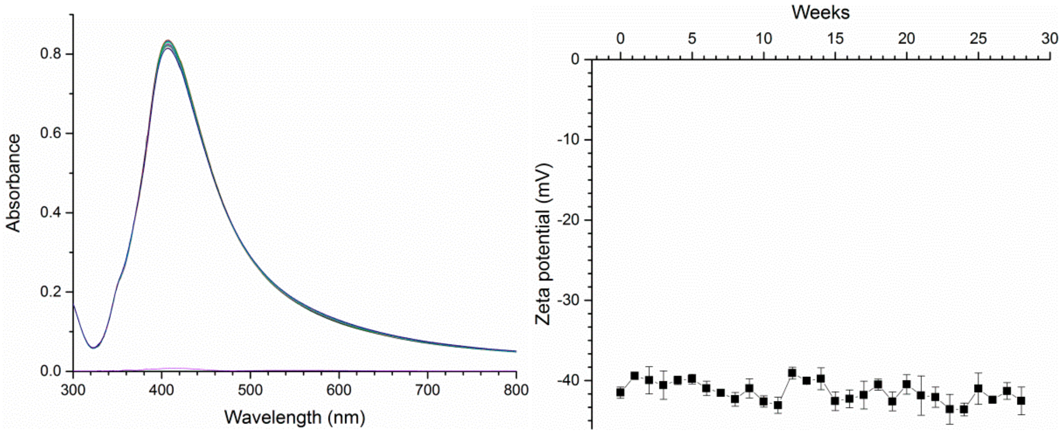

Figure S1. (A) UV/Vis spectra of AgNP colloid over seven months (a total of 28 spectra). (B) Zeta potential of AgNP over seven months. Error bars represent standard deviations of three measurements

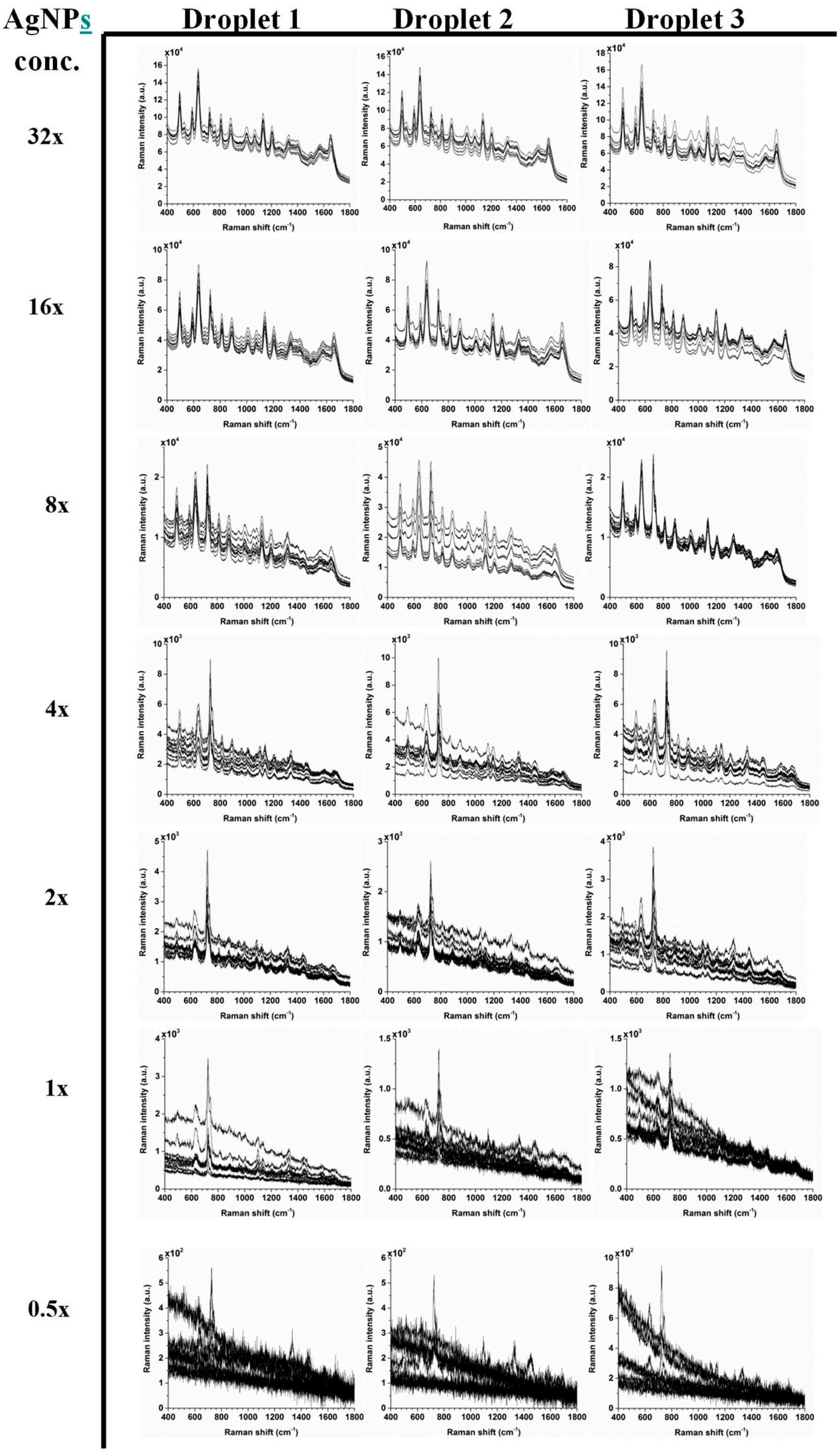

Figure S2. SERS spectra of three dried droplets of whole serum (not filtered) samples with increasing AgNP concentrations

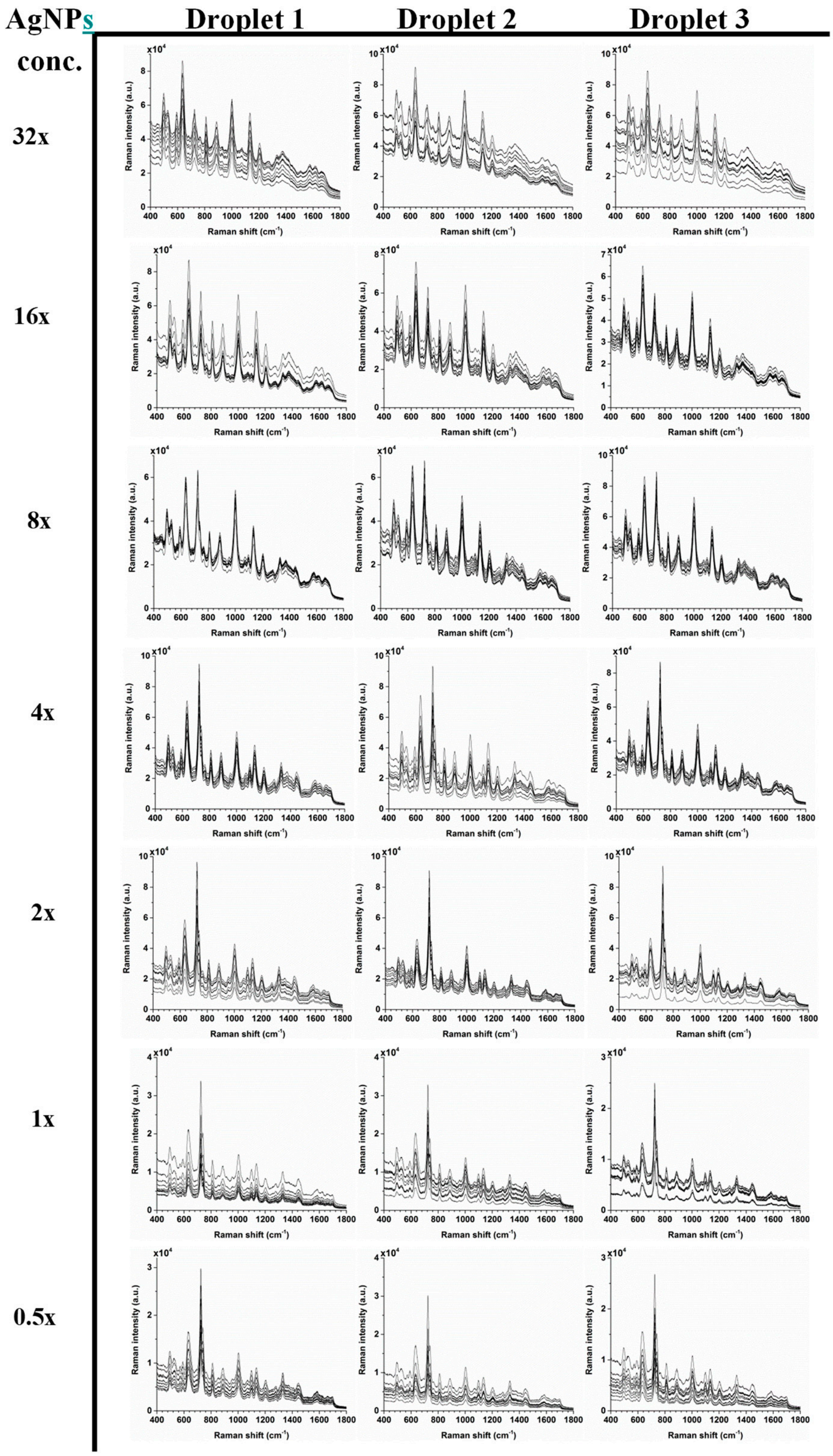

Figure S3. SERS spectra of three dried droplets of filtered serum samples with increasing AgNP concentrations

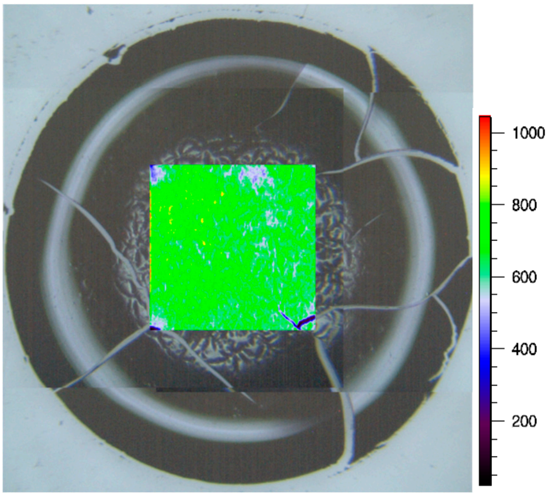

Figure S4. Mapping the middle of a dried droplet. Heat map of the intensity of 638 cm^{-1}.

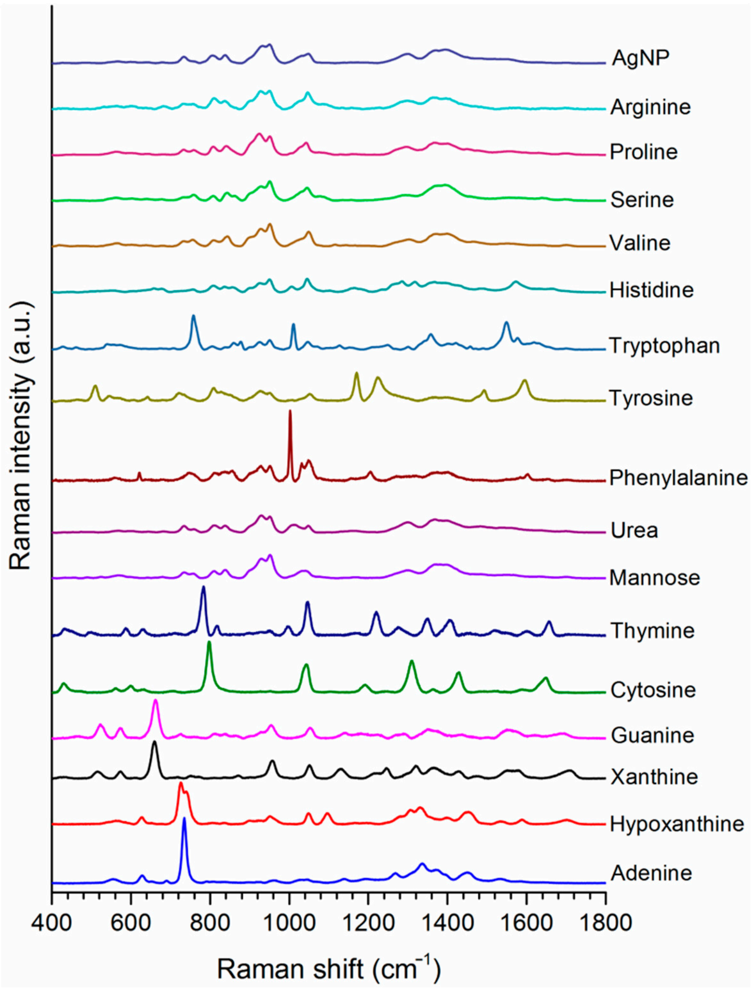

Figure S5. SERS spectra of some molecules found in blood serum and SERS spectrum of AgNPs.

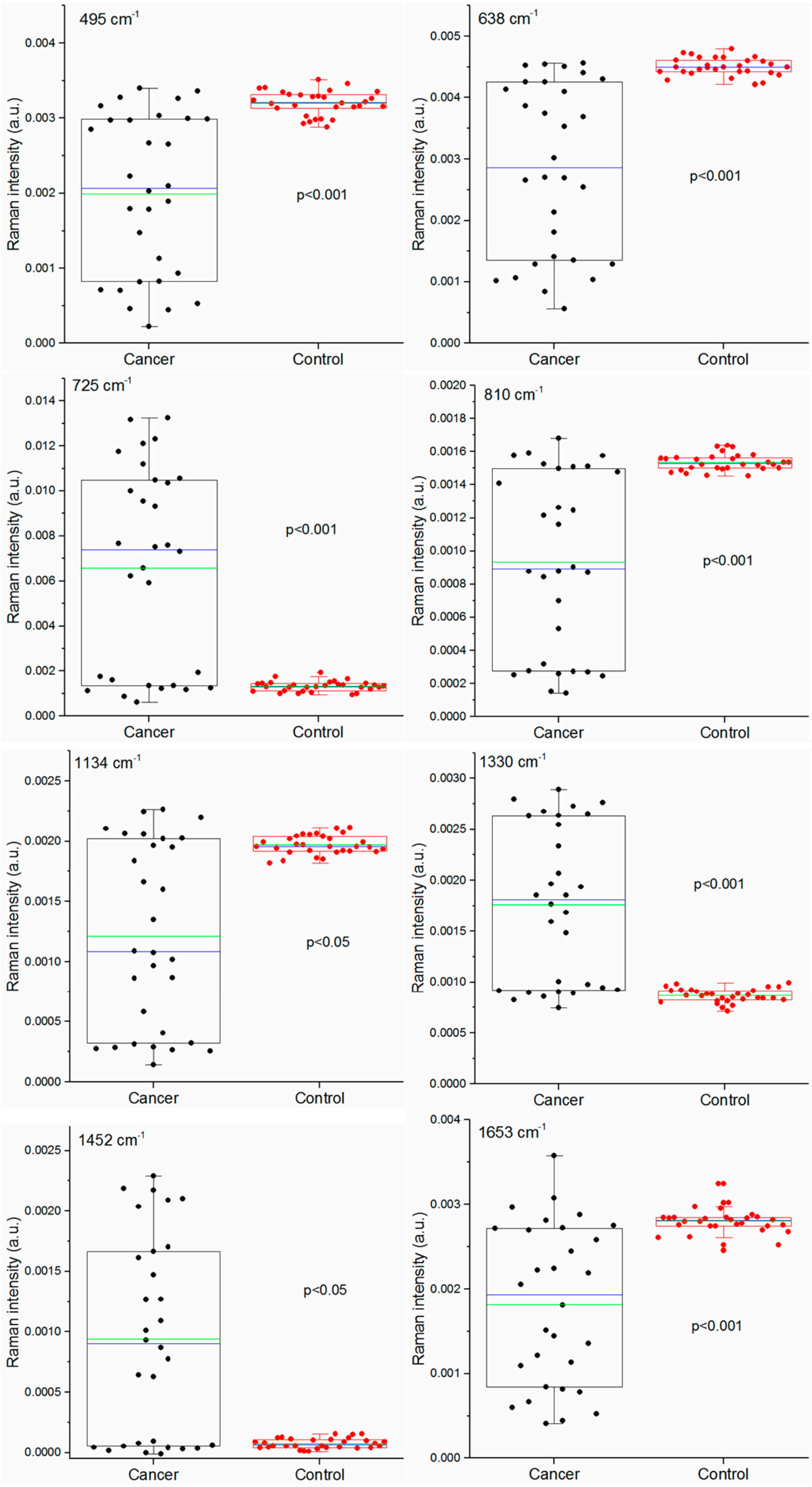

Figure S6. Box plots of the intensity values of the eight significant SERS bands. The blue line and the green line within each box represent median and mean, respectively. Whiskers represent the 1.5-fold interquartile range.

Table S1. Demographics of the cancer patients

Serum samples after tumor removal were obtained from the pink labelled patients. Days passed after tumor removal for second blood take are also shown. NR: Not reported

Patient	Age	Gender	Cancer Type	Days passed after tumor removal for second blood take
P1	55	M	Abdominal cancer	
P2	46	F	Breast cancer	123
P3	57	F	Breast cancer	
P4	73	F	Breast cancer	
P5	60	M	Colon cancer	
P6	61	M	Colon cancer	
P7	66	M	Colon cancer	14
P8	71	M	Colon cancer	58
P9	72	M	Colon cancer	
P10	82	M	Colon cancer	
P11	56	M	Fibrosis	98
P12	66	M	Gastric cancer	21
P13	71	M	Gastric cancer	
P14	56	F	Gastrointestinal cancer	
P15	60	F	Liver cancer	17
P16	63	M	Small intestinal cancer	
P17	65	M	Anorectal cancer	
P18	43	F	Rectal cancer	
P19	51	F	Rectal cancer	
P20	58	F	Rectal cancer	73
P21	41	M	Rectal cancer	13
P22	56	M	Rectal cancer	118
P23	59	M	Rectal cancer	
P24	64	M	Rectal cancer	28
P25	65	M	Rectal cancer	28
P26	66	M	Rectal cancer	90
P27	72	M	Rectal cancer	61
P28	73	M	Rectal cancer	16
P29	77	M	Rectal cancer	
P30	87	M	Rectal cancer	NR
Mean age	63.1			
± SD	10.6			
Median age	63.5			

Table S2. Demographics of patients with chronic diseases.

Patient	Age	Gender	Chronic disease
P1	29	M	heart surgery
P2	38	M	kidney stone
P3	48	M	diabetes
P4	63	M	hypertension
P5	38	F	stomach related
P6	42	F	bronchitis, heart related problems
P7	55	F	hypertension, respiratory distress
P8	55	F	hypertension
P9	55	F	diabetes, hypertension, atherosclerosis
P10	55	F	kidney stone, diabetes
P11	60	F	osteoporosis
P12	63	F	bile stone
P13	66	F	diabetes, hypertension, osteoporosis
P14	66	F	sinuses, osteoporosis
P15	75	F	arthritis
P16	80	F	arthritis
Mean age	55.5		
± SD	13.9		
Median age	55		

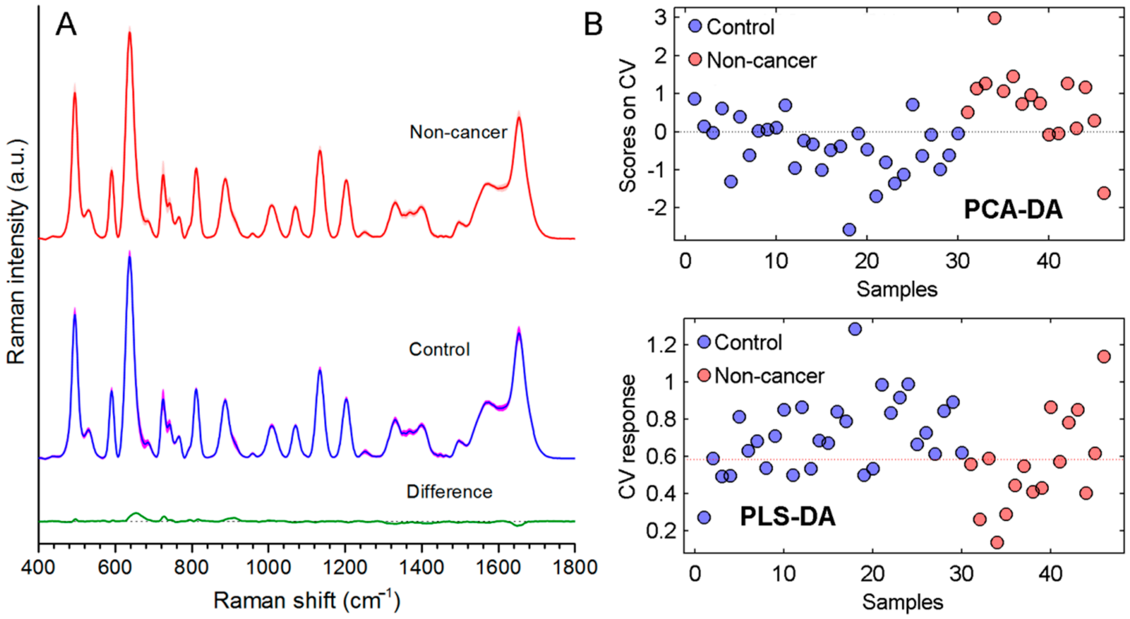

Figure S7. (A) SERS spectra of serum samples of patients with chronic disease (Non-cancer), of healthy subjects (Control) and the difference spectrum of two groups. (B) PCA-DA and PLS-DA scores of each spectrum.

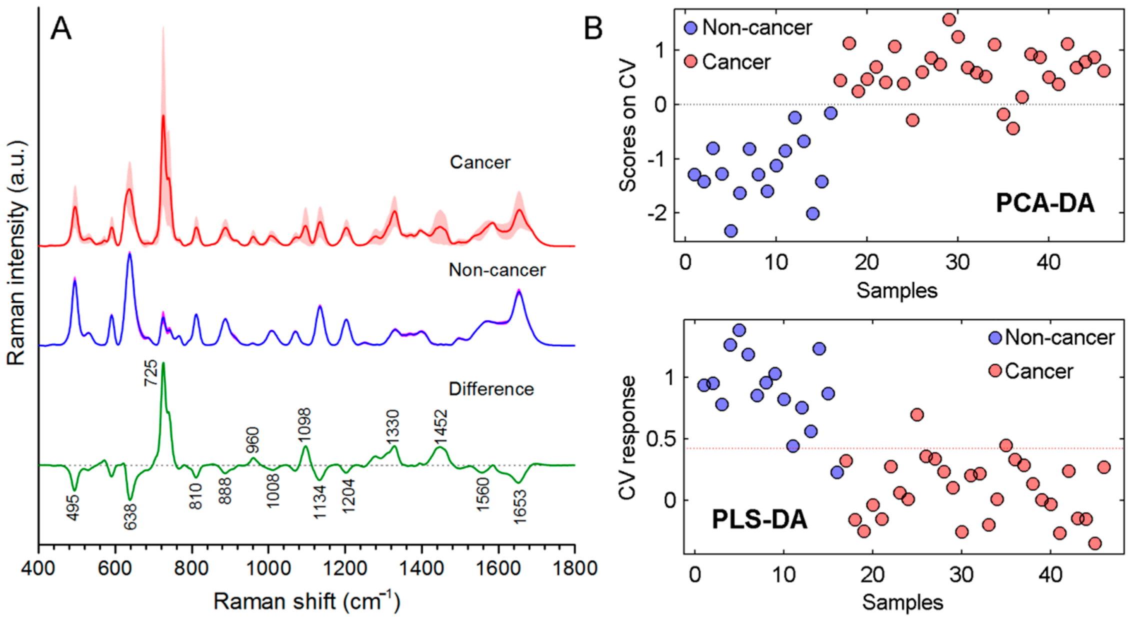

Figure S8. (A) SERS spectra of serum samples of patients with chronic disease (Non-cancer), of cancer patients (Cancer) and the difference spectrum of two groups. (B) PCA-DA and PLS-DA scores of each spectrum.
